# Validity, reliability and applicability of Portuguese versions of sedation-agitation scales among critically ill patients

**DOI:** 10.1590/S1516-31802008000400003

**Published:** 2008-07-03

**Authors:** Antonio Paulo Nassar, Ruy Camargo Pires Neto, Walquiria Barcelos de Figueiredo, Marcelo Park

**Keywords:** Patient monitoring, Sedatives, Psychomotor agitation, Critical care, Reliability, validity, Monitorização fisiológica, Hipnóticos e sedativos, Agitação psicomotora, Cuidados críticos, Reprodutibilidade dos testes

## Abstract

**CONTEXT AND OBJECTIVE:**

Sedation scales are used to guide sedation protocols in intensive care units (ICUs). However, no sedation scale in Portuguese has ever been evaluated. The aim of this study was to evaluate the validity and reliability of Portuguese translations of four sedation-agitation scales, among critically ill patients: Glasgow Coma Score, Ramsay, Richmond Agitation-Sedation Scale (RASS) and Sedation-Agitation Scale (SAS).

**DESIGN AND SETTING:**

Validation study in two mixed ICUs of a university hospital.

**METHODS:**

All scales were applied to 29 patients by four different critical care team members (nurse, physiotherapist, senior critical care physician and critical care resident). We tested each scale for interrater reliability and for validity, by correlations between them. Interrater agreement was measured using weighted kappa (k) and correlations used Spearman's test.

**RESULTS:**

136 observations were made on 29 patients. All scales had at least substantial agreement (weighted κ 0.68-0.90). RASS (weighted κ 0.82-0.87) and SAS (weighted κ 0.83-0.90) had the best agreement. All scales had a good and significant correlation with each other.

**CONCLUSIONS:**

All scales demonstrated good interrater reliability and were comparable. RASS and SAS showed the best correlations and the best agreement results in all professional categories. All these characteristics make RASS and SAS good scales for use at the bedside, to evaluate sedation-agitation among critically ill patients in terms of validity, reliability and applicability.

## RESUMO

**CONTEXTO E OBJETIVO:**

Escalas de sedação são usadas para guiar protocolos de sedação em unidades de terapia intensiva. Entretanto, nenhuma escala em português foi avaliada. O objetivo foi avaliar, quanto a validade e confiabilidade, quatro escalas de sedação/agitação (Glasgow, Ramsay, Richmond Agitation-Sedation Scale, RASS, e Sedation-Agitation Scale, SAS) traduzidas ao português em pacientes de terapia intensiva.

**TIPO DE ESTUDO E LOCAL:**

Estudo de validação em duas UTIs de hospital universitário.

**MÉTODOS:**

Todas as escalas foram aplicadas a 29 pacientes por quatro membros da equipe multiprofissional (uma enfermeira, um fisioterapeuta, um médico intensivista e um residente de medicina intensiva). Cada escala foi testada para confiabilidade interobservador e para validade, usando-se a correlação entre elas. A concordância foi medida pelo *kappa* ponderado e as correlações foram feitas pelo teste de Spearman.

**RESULTADOS:**

Todas as escalas tiveram uma concordância substancial (κ ponderado 0,68-0,90). As escalas RASS (κ ponderado 0,82-0,87) e SAS (κ ponderado 0,83-0,90) tiveram a melhor concordância. Todas as escalas tiveram concordância boa e significante entre elas.

**CONCLUSÕES:**

Todas as escalas tiveram boa concordância interobservador e foram comparáveis entre elas. As escalas RASS e SAS tiveram a melhor correlação entre elas e os melhores resultados de concordância entre as categorias multiprofissionais. Estas características fazem com que as escalas RASS e SAS sejam boas para a avaliação de sedação e agitação de pacientes críticos em termos de validade, confiabilidade e aplicabilidade.

## INTRODUCTION

Analgesic and sedative agents are important tools for managing critically ill patients. During their stay in the intensive care unit (ICU), patients are subjected to painful procedures like tracheal intubation, insertion of catheters and tracheal aspiration.^1^ For comfort during these procedures, the use of analgesics and sedatives is recommended.^2^ However, these agents carry potential risks, such as increased incidence of delirium^3^ and increased time on mechanical ventilation.^4,5^

Sedation protocols are associated with reduced time on mechanical ventilation and with fewer adverse events from sedative drugs.^2,5,6^ With this aim, the guidelines recommend periodic evaluation of sedation levels.^2^ Scales are commonly used to guide sedation levels in ICUs as part of many protocols. There are many sedation scales, but few have been validated or evaluated for reliability and applicability. A systematic review concluded that only four sedation scales developed for adult patients had been adequately evaluated: Glasgow Coma Scale (GCS), Ramsay scale, Sedation and Agitation Scale (SAS), and Motor Activity Assessment Scale (MAAS).^7^ Subsequently, a more recently developed scale was also validated (Richmond Agitation-Sedation Scale, RASS).^8,9^

The clinical usefulness of each instrument should be assessed according to a rational evaluation of its validity, reliability and applicability.^10^ Validity is the ability of a tool to actually measure the parameter that it is designed for. In monitoring sedation, this concept implies the ability to document agitation and distress symptoms (anxiety, delirium and pain), as well as identifying the endpoints of each level of sedation that each sedative agent can achieve. Reliability is the capacity of a test to obtain similar measurements with different observers. Applicability in this context implies that an instrument is easy to learn and operate, and that it is suitable for routine use by physicians, physiotherapists and nurses.^7,10^

Although sedation-agitation scales are commonly used in Brazilian ICU practice, to the best of our knowledge there is no report evaluating the clinical usefulness of these scales in the Portuguese language. The commonly used sedation-agitation scales in ICU practice are GCS, Ramsay, SAS and RASS,^2^ and all of these have been tested for validity, reliability and applicability in the English language.^7,8,11^ Such evaluations are important: they are able to demonstrate that these scales can be useful in sedation protocols and in initiatives aimed at changing practices and reducing morbidity in ICUs.

## OBJECTIVE

In order to assure the clinical usefulness of sedation-agitation scales for routine practice in a Brazilian ICU, the aim of this study was to evaluate the validity, reliability and applicability of Portuguese translations of four sedation scales (GCS, Ramsay, SAS and RASS).

## MATERIALS AND METHODS

This study was conducted in two mixed medical-surgical ICUs (25 beds). All patients admitted to these units on two consecutive days were evaluated by four members of the multidisciplinary team (a nurse, a physiotherapist, a senior critical care physician and a critical care resident), using four sedation scales translated into Portuguese (Appendix – Panels 1 to 4).

Patients with hearing deficits and those who did not speak Portuguese would have been excluded according to the study protocol, but there were no patients in these categories on the study days.

No interventions were made regarding the patients’ treatment, and no adjustments were made to the sedation and analgesic drugs that were being administered. All patients under continuous sedation were receiving a combination of midazolam (150 mg of midazolam diluted in 120 ml of saline solution, giving a final concentration of 1 mg/ml) and fentanyl (50 ml pure, in a burette), in different venous infusion pumps. No sedation protocol was used in the ICUs, and the sedation doses were at the discretion of the attending physicians. Changes were implemented by the nursing team following verbal orders from physicians.

The study was approved by the local Ethics Committee and informed consent was waived.

### Study protocol

**Figure 1:**
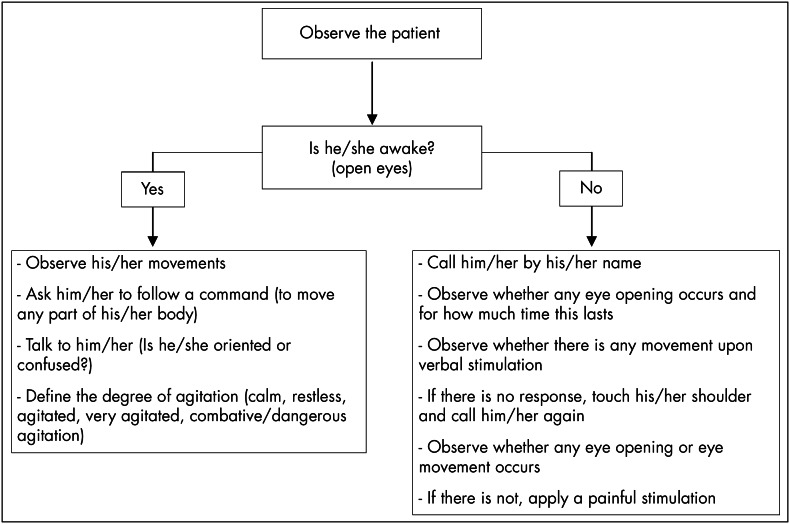
Study protocol for evaluating sedation-agitation levels.

All four members underwent a period of training, to learn how to use the four scales. All had previous knowledge of GCS and the two physicians had some practical experience with Ramsay and SAS. After this training, there was a pilot study with four patients, in which each of the investigators applied the four scales using defined methodology and had the opportunity to discuss rates and difficulties in applying them. During the study, all patients were evaluated using predefined methodology (Figure 1). For each evaluation, a different investigator interacted with the patient, but all four investigators gave a rate for each scale.

We collected the following data from the patients: age, gender, reason for admission (medical or surgical), Acute Physiology and Chronic Health Evaluation II (APACHE II) and use of invasive mechanical ventilation.

### Validity and reliability

Validity can be defined as the ability of an instrument to measure what it is intended to. Since there is no reliable method for measuring level of consciousness or agitation, we decided to test the sedation scales against each other and against GCS. This is the way that other studies have found to validate such scales.^8,11^ Reliability is defined as the capacity to get similar scores between different raters. To test reliability, we measured the concordance between the four investigators, two by two.^7,10^

### Statistical analyses

Continuous variables are presented as medians and interquartile ranges, except for drug doses, which are presented as means and standard deviations. Category variables are presented as frequencies and percentages. Interrater reliability was determined for RASS, Ramsay, SAS and GCS by comparing ratings between the investigators, using weighted kappa (κ) indices and 95% confidence intervals. To evaluate the validity, all scores were compared with each other, two by two, using the Spearman correlation coefficient (r). Statistical analyses were performed using the Statistical Package for the Social Sciences (SPSS) version 10.0 and Medical Calculator (MedCalc) version 9.0.

## RESULTS

A total of 29 patients were eligible for the study. The baseline characteristics are presented in Table 1. The patients who were under continuous sedation received midazolam and fentanyl at mean doses of 4.8 ± 3.3 mcg/kg/h and 0.12 ± 0.07 mg/kg/h respectively. The patients were evaluated on two days during the afternoon. A total of 136 scores were available from each scale. SAS and RASS had the highest interrater agreement, but all comparisons had at least a substantial agreement (> 0.60) (Table 2). The interrater reliability of RASS and SAS was very good (> 0.80) across all members of the multidisciplinary team.

**Table 1:** Baseline characteristics of the patients

Characteristic	Number
Age (years)*	63 [48 - 68]
Female sex, n (%)	19 (70)
Reason for admission	
Medical, n (%)	17 (58.6)
Surgical, n (%)	10 (34.5)
Trauma, n (%)	2 (6.9)
APACHE II*	18 [12 - 19]
Mechanical ventilation, n (%)	10 (35)
Continuous sedation, n (%)	5 (17)

* *Data are shown as medians and interquartile ranges;*

*APACHE = acute physiology and chronic health evaluation.*

**Table 2:** Interrater reliability of the four sedation-agitation scales

	RASS weighted κ (95% CI)	GCS weighted κ (95% CI)	Ramsay weighted κ (95% CI)	SAS weighted κ (95% CI)
Nurse versus physiotherapist	0.86 (0.77-0.94)	0.81 (0.67-0.94)	0.83 (0.71-0.94)	0.83 (0.67-0.97)
Nurse versus physician	0.89 (0.81-0.97)	0.72 (0.56-0.89)	0.82 (0.69-0.96)	0.89 (0.77-0.99)
Nurse versus resident	0.85 (0.77-0.94)	0.86 (0.74-0.98)	0.75 (0.61-0.89)	0.86 (0.73-0.99)
Physiotherapist versus physician	0.87 (0.79-0.96)	0.82 (0.68-0.95)	0.68 (0.50-0.87)	0.87 (0.74-0.99)
Physiotherapist versus resident	0.86 (0.78-0.94)	0.86 (0.76-0.95)	0.78 (0.64-0.92)	0.90 (0.80-0.99)
Physician versus resident	0.82 (0.73-0.92)	0.73 (0.56-0.89)	0.82 (0.70-0.94)	0.90 (0.78-0.99)

*CI = confidence interval; κ = kappa; RASS = Richmond agitation-sedation scale; SAS = sedation-agitation scale; GCS = Glasgow Coma Scale.*

There was a significant (p < 0.001) and at least moderate (r > 0.7 or < -0.7) correlation among all scales tested. The strongest correlation was between SAS and RASS (Table 3). We did not conduct any subgroup analyses because we considered that our sample was small and all such analyses would lack statistical power.

**Table 3:** Spearman correlation coefficients between scales

	Correlation (r)	p-value
GCS versus RASS	0.70	< 0.001
GCS versus SAS	0.74	< 0.001
GCS versus Ramsay	- 0.82	< 0.001
RASS versus SAS	0.91	< 0.001
RASS versus Ramsay	- 0.79	< 0.001
SAS versus Ramsay	- 0.85	< 0.001

*GCS = Glasgow Coma scale; RASS = Richmond agitation-sedation scale; SAS = sedation-agitation scale.*

## DISCUSSION

To the best of our knowledge, this was the first study evaluating sedation-agitation scales in Portuguese. Our results showed that all of the four scales evaluated (GCS, Ramsay, SAS and RASS) had substantial interrater agreement and at least a moderate correlation.

It is recommended that sedation should be routinely assessed among critically ill patients,^2^ but this is not a common practice. A national survey in Canada showed that only 49% of the intensive care specialists used a sedation scoring system.^12^ Another study in 44 ICUs in France showed that only 43% of patients were evaluated for sedation and only 42% were evaluated for analgesia by the second day in the ICU.^13^ This lack of routine assessment has potentially harmful consequences. Oversedation is associated with increased duration of mechanical ventilation and all of its consequences.^4^ Recently, it has been shown that sedatives are also associated with increased incidence of delirium,^3^ and probably with posttraumatic stress disorder.^14^ Oversedation and delirium can also interfere in pain evaluations among critically ill patients, while pain is the most important stress factor during ICU stay.^15^ The positive impact of systematically evaluating pain and agitation in ICUs has recently been demonstrated. Such evaluations led to fewer patients reporting pain, lower incidence of severe agitation, reduced duration of mechanical ventilation and reduced incidence of nosocomial infections.^16^ Therefore, it is very important to routinely assess sedation among critically ill patients, and sedation-agitation scales are instruments that make it possible to achieve appropriate sedation.^2^

The GCS is a coma scale, and its use has been extrapolated to sedation quantification.^12,13^ Thus, it is not expected to measure agitation adequately. For this reason, the correlation we found between the GCS and other scales was not good (Table 3). On the other hand, it is widely known and used, and the agreement between observers should be high. However, this was not shown in our results, probably because of the absence of precise definitions for rating the scale.

Among sedation scales, the Ramsay scale is the one that is most used in ICU practice.^12,13^ It is the oldest scale and the one most used in clinical studies.^5^ It is a scale that is able to identify somnolence and agitation visually. However, some authors have suggested that Ramsay's sedation levels are not conclusive.^10^ In our study, Ramsay was the scale that showed the worst agreement with the other ones. A systematic review showed that the interrater agreement on the Ramsay scale was between 0.79 and 0.87, and those results were superior to ours.^7^ This may have been because the observers involved in the studies included in that review had had greater practice with this scale than had our observers. However, it is noteworthy that Ramsay presented lower interrater agreement than did the sedation scales to which it was compared.^7^ We would like to stress that Ramsay's score items are not clearly defined and doubts can rise when using that scale. It is also noteworthy that the agreement between the physicians in our study was not good, even though they had been expected to present the best agreement because they had had previous knowledge and practice with that scale. This indicates difficulties in conceptual definitions when choosing an item on Ramsay's scale.

In our study, the SAS and RASS scales had the best agreement among the observers. These are newer scales that also have the ability to define agitation levels.^8,11^ When evaluating sedation levels, both of them systematically differentiate verbal and physical stimulation, and this characteristic makes it easy to choose a score during the evaluation. RASS has also step-by-step methodology for applying it, and this has probably contributed towards choosing it as the scale used in newer studies. Although not all the investigators had had practice with these two scales, their agreement was almost perfect. Other studies have already shown that SAS and RASS have at least substantial agreement (0.92 for SAS^11^ and values ranging from 0.64 to 0.91 for RASS).^8,9^ These data indicate that RASS and SAS are easy to apply at the bedside.

It is important to emphasize that these scales are used to evaluate not only sedation levels but also agitation levels. Therefore, they are commonly applied to patients without intubation in almost all validation studies, and they serve as screening tools for evaluating delirium.^3^ In our study, 35% of the patients were intubated. This was not different from other validation studies, in which intubated patients accounted for between 35 and 100% of all patients.^8,9,11,17,18^

Our study has some limitations. Firstly, the investigators were only trained for a short time before applying the sedation scales. Perhaps if our training had been better, the interrater agreement could have been greater even for the Ramsay scale. A “learning curve” seems to exist, in that all studies that have evaluated a scale for the second time had better interrater agreement than the first ones. Secondly, we conducted this study with only 29 patients. Other validation studies have used a greater number of patients.^8,9,17,18^ However, our study included 136 observations made by four different members of the multidisciplinary team. Only the RASS validation studies in English were conducted with four or more different individuals applying a scale.^8,9^ In all other validation studies, sedation scales were evaluated with two investigators^11,17^ and in only one case, with three investigators.^18^

## CONCLUSIONS

In conclusion, the Portuguese versions of GCS, Ramsay, RASS and SAS presented substantial agreement between raters and significant correlations with each other. RASS and SAS showed the best correlation and the best agreement results in all professional categories. All these characteristics make RASS and SAS good scales for use at the bedside to evaluate sedation-agitation among critically ill patients in terms of validity, reliability and applicability. These two scales can be used in clinical practice, protocol sedations and interventions with the aim of reducing the negative impacts of oversedation and agitation.

## APPENDIX. Portuguese translations of four sedation-agitation scales: Ramsay, Sedation-agitation scale (SAS), Richmond agitation-sedation scale (RASS) and Glasgow Coma Score (GCS)

**Panel 1:** Ramsay sedation scale

**Acordado**	
1	Ansioso e/ou agitado.
2	Cooperativo, orientado e tranqüilo.
3	Obedece a comandos.
**Dormindo**	
4	Tranqüilo, pronta resposta à percussão glabelar ou estímulo sonoro.
5	Resposta lentificada à percussão glabelar ou estímulo sonoro.
6	Sem resposta.

**Panel 2:** Sedation-agitation scale (SAS)

7	Agitação perigosa: tentativa de retirar tubo orotraqueal ou cateter ou de sair da cama, agredir a equipe, movimento de um a outro lado da cama.
6	Muito agitado: morde o tubo, necessidade de restrições, não se acalma com orientação verbal com estabelecimento de limites.
5	Agitado: ansioso ou levemente agitado, tentando levantar, acalma com orientação verbal.
4	Calmo e cooperativo: calmo, acorda fácil, obedece a comandos.
3	Sedado: difícil de acordar, acorda com estímulo verbal ou gentil chacoalhar, mas volta a dormir. Obedece a comandos simples.
2	Muito sedado: acorda com estímulo físico, mas não responde ordens. Move-se espontaneamente.
1	Não despertável: resposta mínima ou não responde a estímulos ou ordens. Não se comunica.

**Panel 3:** Richmond agitation-sedation scale (RASS)

Pontos	Termo	Descrição
+ 4	Combativo	Claramente combativo, violento, representando risco para a equipe
+ 3	Muito agitado	Puxa ou remove tubos ou cateteres, agressivo verbalmente
+ 2	Agitado	Movimentes despropositados freqüentes, briga com o ventilador
+ 1	Inquieto	Apresenta movimentos, mas que não são agressivos ou vigorosos
0	Alerta e calmo	
- 1	Sonolento	Adormecido, mas acorda ao ser chamado (estímulo verbal) e mantém os olhos abertos por mais de 10 segundos
- 2	Sedação leve	Despertar precoce ao estímulo verbal, mantém contato visual por menos de 10 segundos
- 3	Sedação moderada	Movimentação ou abertura ocular ao estímulo verbal (mas sem contato visual)
- 4	Sedação intensa	Sem resposta ao ser chamado pelo nome, mas apresenta movimentação ou abertura ocular ao toque (estímulo físico)
- 5	Não desperta	Sem resposta ao estímulo verbal ou físico

**Panel 4:** Glasgow Coma Score (GCS)

**Abertura ocular**
Sem abertura Abertura ao estímulo doloroso Abertura ao chamado Abertura espontânea
**Resposta verbal**
Sem resposta Sons incompreensíveis Palavras ou frases sem sentido Confusão mental Orientado têmporo-espacialmente
**Resposta motora**
Sem resposta Descerebração (extensão de membros superiores e inferiores) Decorticação (Flexão de cotovelos e punhos e extensão de membros inferiores) Resposta inespecífica à dor Localização e movimento de retirada ao estímulo doloroso Obedece a comandos simples
